# Aβ reduction in BACE1 heterozygous null 5XFAD mice is associated with transgenic APP level

**DOI:** 10.1186/1750-1326-10-1

**Published:** 2015-01-07

**Authors:** Katherine R Sadleir, William A Eimer, Sarah L Cole, Robert Vassar

**Affiliations:** Department of Cell and Molecular Biology, Feinberg School of Medicine, Northwestern University, Chicago, IL 60605 USA; Department of Neurology, Massachusetts General Hospital, Charlestown, MA 02129 USA; Takeda Cambridge, Neurodegeneration Group, Cambridge, UK

**Keywords:** β-secretase, Amyloid precursor protein, Alzheimer’s disease, APP transgenic mouse models, 5XFAD, BACE1 heterozygous, Amyloid, Aβ, Dot blot, Estrogen response element

## Abstract

**Background:**

The β-secretase, BACE1, cleaves APP to initiate generation of the β-amyloid peptide, Aβ, that comprises amyloid plaques in Alzheimer’s disease (AD). Reducing BACE1 activity is an attractive therapeutic approach to AD, but complete inhibition of BACE1 could have mechanism-based side-effects as BACE1^−/−^ mice show deficits in axon guidance, myelination, memory, and other neurological processes. Since BACE1^+/−^ mice appear normal there is interest in determining whether 50% reduction in BACE1 is potentially effective in preventing or treating AD. APP transgenic mice heterozygous for BACE1 have decreased Aβ but the extent of reduction varies greatly from study to study. Here we assess the effects of 50% BACE1 reduction on the widely used 5XFAD mouse model of AD.

**Results:**

50% BACE1 reduction reduces Aβ42, plaques, and BACE1-cleaved APP fragments in female, but not in male, 5XFAD/BACE1^+/−^ mice. 5XFAD/BACE1^+/+^ females have higher levels of Aβ42 and steady-state transgenic APP than males, likely caused by an estrogen response element in the transgene Thy-1 promoter. We hypothesize that higher transgenic APP level in female 5XFAD mice causes BACE1 to no longer be in excess over APP so that 50% BACE1 reduction has a significant Aβ42 lowering effect. In contrast, the lower APP level in 5XFAD males allows BACE1 to be in excess over APP even at 50% BACE1 reduction, preventing lowering of Aβ42 in 5XFAD/BACE1^+/−^ males. We also developed and validated a dot blot assay with an Aβ42-selective antibody as an accurate and cost-effective alternative to ELISA for measuring cerebral Aβ42 levels.

**Conclusions:**

50% BACE1 reduction lowers Aβ42 in female 5XFAD mice only, potentially because BACE1 is not in excess over APP in 5XFAD females with higher transgene expression, while BACE1 is in excess over APP in 5XFAD males with lower transgene expression. Our results suggest that greater than 50% BACE1 inhibition might be necessary to significantly lower Aβ, given that BACE1 is likely to be in excess over APP in the human brain. Additionally, in experiments using the 5XFAD mouse model, or other Thy-1 promoter transgenic mice, equal numbers of male and female mice should be used, in order to avoid artifactual gender-related differences.

## Background

Alzheimer’s disease (AD) is characterized by two types of pathological lesions: neurofibrillary tangles, which are composed of hyperphosphorylated forms of the protein Tau, and amyloid plaques, which consist of aggregations of the peptide β-amyloid (Aβ). Cerebral accumulation of Aβ, especially the 42-amino acid isoform Aβ42, is thought to play a critical early role in the development of AD [1, 2], so there is great interest in decreasing Aβ levels in the brain to prevent or treat AD. Aβ is generated by the sequential cleavage of Amyloid Precursor Protein (APP) by β-secretase (BACE1) and γ-secretase. APP transgenic mouse models of AD that are devoid of BACE1 expression via BACE1 gene knockout (BACE1^−/−^ mice) fail to generate Aβ and lack the amyloid plaques and cognitive impairments found in APP transgenic mice that express both BACE1 alleles [3–7]. These results validated BACE1 as the major β-secretase enzyme in the brain and suggested that inhibition of BACE1 could be of therapeutic benefit for AD (reviewed in [8]). Indeed, BACE1 inhibitor drugs are currently being tested in clinical trials in AD and mild cognitively impaired individuals (reviewed in [9]). However, the long-term safety and efficacy of BACE1 inhibitor drugs are unknown at present.

Initially, BACE1 null mice were reported to be healthy with no obvious phenotype [5, 10], but more extensive analysis revealed that they have subtle neurological abnormalities [8, 9]. Subsequent studies have found that BACE1 null mice have higher offspring mortality, decreased myelination, impaired memory, hyperactivity, axon mis-guidance, schizophrenia-like phenotypes, and increased seizure activity, but these phenotypes are largely absent from BACE1^+/−^ mice [6, 7, 11–18]. The BACE1 substrates responsible for some of these phenotypes are known, such as the role of neuregulin in myelination [13, 17] and CHL1 in axon guidance [18], but others remain unexplained. Proteomic screens of BACE1^−/−^ compared to BACE1^+/+^ primary neurons [19, 20] have revealed even more potential BACE1 substrates that are not yet validated *in vivo* but could have a role in these phenotypes, and others yet to be described.

Since complete loss of BACE1 activity has detrimental effects in BACE1^−/−^ mice it seems likely that almost complete inhibition of BACE1 for treatment or prevention of Alzheimer’s disease could have mechanism based side-effects in humans. The 50% BACE1 reduction observed in in BACE1^+/−^ mice, on the other hand, seems to have no ill effects. If 50% inhibition of BACE1 is able to decrease Aβ production enough to delay disease onset or slow disease progression, this could represent a therapeutic strategy to avoid side effects of almost total BACE1 inhibition. The BACE1^+/−^ heterozygous null mouse is a useful model for 50% BACE1 inhibition, and several publications have described BACE1^+/−^ mice on various backgrounds of APP transgenic mouse models, with most observing some reduction in Aβ levels, but the degree of Aβ lowering varies from model to model [5, 14, 21–26]. It is also unclear whether 50% reduction in BACE1 leads to a long-lasting decrease in cerebral Aβ. It has been reported in the PDAPP mouse model that BACE1^+/−^ genotype led to a small reduction in Aβ at 3 months of age, but dramatic Aβ decreases at 13 and 18 months [24]. On the other hand, in transgenic mice co-expressing APP Swedish (swe) and presenilin 1 exon 9 deletion (PS1Δ9) familial AD (FAD) mutations, BACE1^+/−^ genotype led to decreased cerebral Aβ and plaques at 12 months, but not at 20 months of age [14].

This work extends the study of 50% BACE1 inhibition as a therapeutic approach, demonstrating that 50% BACE1 reduction in 5XFAD transgenic mice, which display aggressive, early onset amyloid pathology [27], decreases Aβ42, plaques, and BACE1-cleaved APP fragments (C99 and sAPPβ) at 4, 6 and 9 months of age, but unexpectedly only in females, which have higher levels of Aβ42 and amyloid plaques than males. Other work reported a reduction in Aβ, amyloid deposition, and amelioration of cognitive deficits in 5XFAD/BACE1^+/−^ mice, but did not differentiate between the sexes [21–23]. We attribute the elevated Aβ42 and amyloid deposition in female 5XFAD to higher levels of APP transgene expression due to an estrogen response element (ERE) found in the Thy-1 promoter of the transgene. The 5XFAD mouse model has become quite widely used in the Alzheimer’s field, and this study highlights the importance of using cohorts of the same gender, or containing equal numbers of each sex. If experimental and control groups are not gender balanced, effects on cerebral Aβ and amyloid pathology may be observed that are not due to experimental manipulation, but to higher Aβ levels in female mice.

We hypothesize that the lower level of expression of the APP transgene in 5XFAD males is the cause of the decreased cerebral Aβ42 and amyloid, and leads to a situation where BACE1 is in excess of APP, even when reduced by 50%, so that no Aβ42 lowering occurs in 5XFAD/BACE1^+/−^ mice. In contrast, because of higher transgenic APP levels in 5XFAD females, BACE1 is not in excess over APP, thus resulting in substantial Aβ42 lowering with 50% BACE1 reduction. These results suggest that 50% BACE1 inhibition would be an effective therapeutic approach to decreasing cerebral Aβ42 levels only under conditions where BACE1 is not in excess over APP. In the case of human AD patients, APP is not overexpressed, and BACE1 is increased during the course of disease, suggesting BACE1 is likely to be present in excess of APP, limiting the therapeutic efficacy of reducing BACE1 activity by 50%. Finally, we also report an accurate, simple, and inexpensive dot blot assay to measure cerebral Aβ42 levels as an alternative to Aβ42 ELISA.

## Results

### BACE1 levels are consistently reduced by ~50% in 5XFAD/BACE1^+/−^ mice at all ages and are the same in both genders

**Figure 1:**
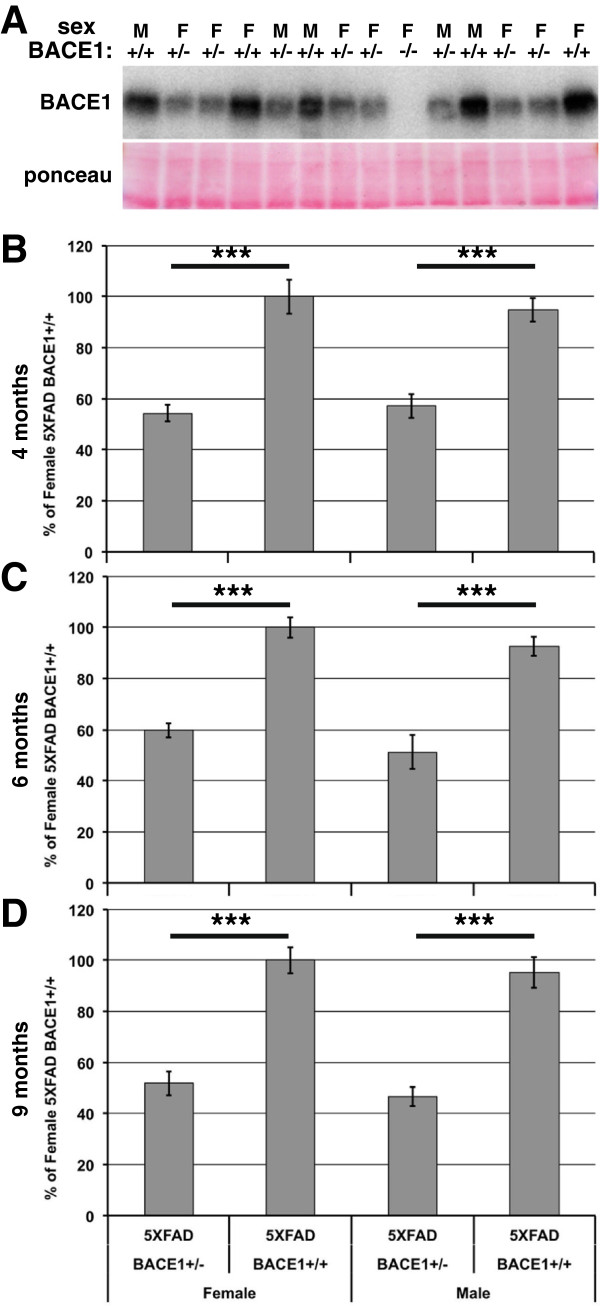
**BACE1 levels are reduced by ~50% in 5XFAD/BACE1** ^**+/−**^ **mice. (A)** 20 μg of protein/lane of brain homogenates of 4, 6, and 9 month-old 5XFAD/BACE1^+/+^, 5XFAD/BACE1^+/−^ or 5XFAD/BACE1^−/−^ (as a negative control) female (F) and male (M) mice were separated by SDS-PAGE, transferred onto PVDF membrane, stained with Ponceau S, and incubated with BACE-Cat1 monospecific antibody against BACE1 [28] followed by HRP-conjugated secondary antibody and chemiluminescence imaging. Representative BACE1 immunoblot and Ponceau S staining of brain homogenates from 4 month-old mice is shown. Note the absence of BACE1 immunosignal in the BACE1^−/−^ lane. **(B-D)** Relative quantification of BACE1 levels in brain homogenates of 4, 6, and 9 month-old female and male 5XFAD/BACE1^+/+^, 5XFAD/BACE1^+/−^ or 5XFAD/BACE1^−/−^ mice. BACE1 immunosignals were normalized to Ponceau S staining intensity per lane, averaged for each group, then presented as percentage of mean female 5XFAD/BACE1^+/+^ BACE1 level. At all ages, BACE1 level is significantly reduced by ~50% in 5XFAD/BACE1^+/−^ mice compared to 5XFAD/BACE1^+/+^ mice, and there is no difference in BACE1 level between male and female 5XFAD/BACE1^+/+^ or male and female 5XFAD/BACE1^+/−^ mice.

Recent studies suggest that complete therapeutic inhibition of BACE1 might be associated with complex neurological mechanism-based side effects (reviewed in [8, 9]). Therefore, partial BACE1 inhibition might offer a safer therapeutic option, provided enough BACE1 inhibition can be achieved to significantly reduce Aβ42 production in the brain. The goals of the present study were to determine 1) the extent to which partial BACE1 inhibition lowers brain Aβ42 levels, 2) establish an accurate cost-effective Aβ42 dot blot assay as an alternative to Aβ42 ELISA. To accomplish these goals, we modeled partial BACE1 inhibition by crossing BACE1^−/−^ mice [3, 5, 11] with 5XFAD transgenic mice that exhibit aggressive, early-onset amyloid pathology [27]. 5XFAD offspring that were BACE1^+/+^, ^+/−^, or ^−/−^ were aged to 4, 6, and 9 months of age and brains analyzed for BACE1, Aβ42, APP, and BACE1-cleaved APP fragments. We analyzed females and males separately in all experiments because our initial characterization of 5XFAD mice [27] and subsequent work showed that females accumulate higher levels of cerebral Aβ42 on average than males. Initially, we measured BACE1 levels in homogenates of hemibrains from these mice by immunoblot analysis using the monospecific anti-BACE1 antibody BACE-Cat1 [28]. As expected, at all ages 5XFAD/BACE1^+/−^ mice have ~50% of the BACE1 level exhibited by 5XFAD/BACE1^+/+^ mice (Figure 1). Moreover, for a given BACE1 genotype, females and males have the same level of BACE1, demonstrating that increased accumulation of cerebral Aβ42 in females is not the result of elevated BACE1. For this analysis, and all subsequent analyses of the 5XFAD/BACE1 mice, we analyzed 6 to 17 animals for each age/genotype/sex combination, as described in detail in the Methods.

### Dot blot with an Aβ42-selective antibody effectively measures Aβ42 levels in 5XFAD brain homogenates

**Figure 2:**
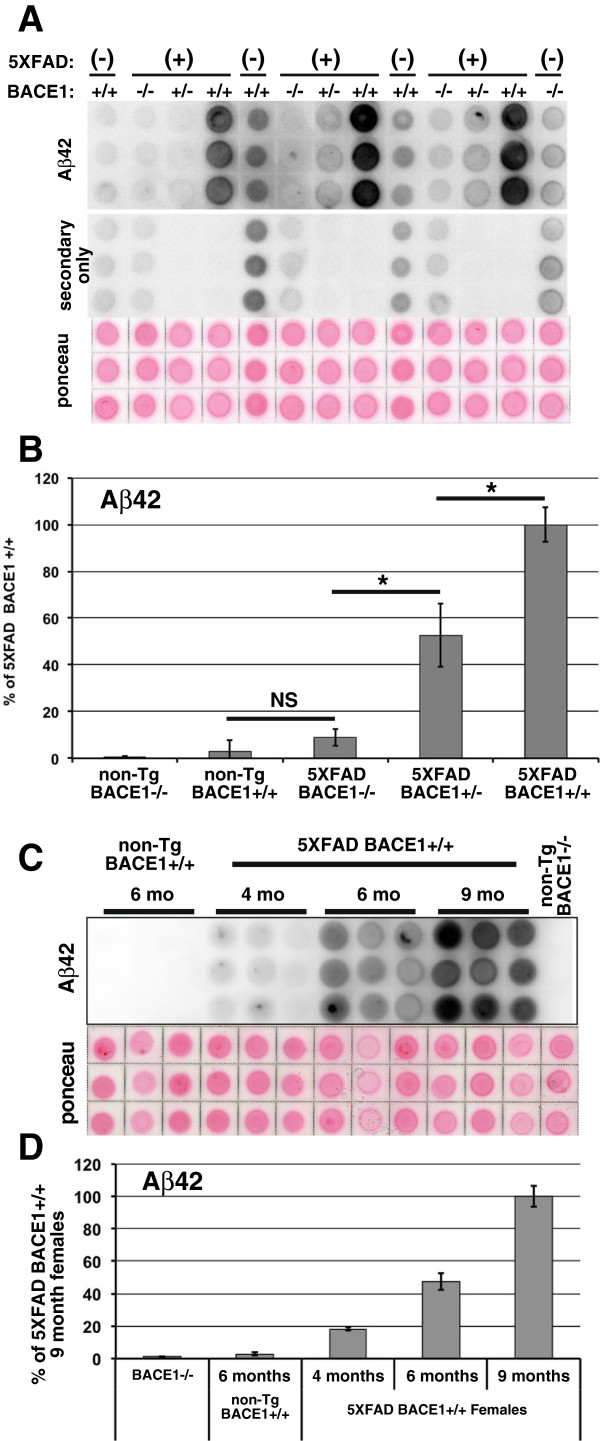
**Dot blot assay effectively measures relative levels of cerebral Aβ42. (A)** Validation of blot assay. Brain homogenates from 6 month-old female mice were extracted overnight in GuHCl, then spotted in triplicate on nitrocellulose membrane, dried, stained with Ponceau S, and incubated with anti-Aβ42 antibody followed by HRP-conjugated secondary antibody and chemiluminescence imaging. To subtract non-specific background, a duplicate membrane was prepared in the same way, except that the anti-Aβ42 primary antibody was not included (secondary only). (+) or (−) indicate 5XFAD or non-transgenic genotypes, respectively; BACE1 genotype is indicated below 5XFAD genotype. **(B)** Relative quantification of the Aβ42 dot blot in **(A)**. The secondary only background was subtracted from the Aβ42 immunosignal, then the difference was normalized to Ponceau S staining intensity and triplicates averaged. Group means were calculated and presented as percentages of the 5XFAD/BACE1^+/+^ female group. The Aβ42 level in 5XFAD/BACE1^−/−^ females is not significantly different than that of non-Tg BACE1^+/+^ females, indicating that the anti-Aβ42 antibody does not recognize full-length APP. 5XFAD/BACE1^+/−^ mice have significantly less Aβ42 than 5XFAD/BACE1^+/+^ mice and significantly more Aβ42 than 5XFAD/BACE1^−/−^ mice. n = 6 per genotype, except n = 2 for non-Tg/BACE1^−/−^. **(C)** Aβ42 dot blot demonstrating the dynamic range of the assay. Brain homogenates from female 4, 6, and 9 month-old 5XFAD/BACE1^+/+^ (n = 6), 6 month-old non-Tg BACE1^+/+^ (n = 6), and BACE1^−/−^ (n = 2) mice were prepared as in **(A)**, except homogenates were extracted in GuHCl for 72 rather than 24 hrs, which eliminated the IgG background. Since there was no secondary only signal, this blot is not shown, or used for correction. **(D)** Relative quantification of the Aβ42 dot blot in **(C)** shows at least a 10-fold dynamic range of detection, and clear differences are observed between groups.

We next endeavored to investigate the effect of a 50% reduction in BACE1 on Aβ42 level in 5XFAD mice. Commercial sandwich ELISAs that measure Aβ42 levels are accurate and sensitive but expensive, thus limiting their use. Therefore, we developed a simple, robust, and accurate Aβ42 dot blot assay as a cost-effective alternative to commercial ELISAs for measuring levels of Aβ42 in APP transgenic mouse and human AD brain. To validate the Aβ42 dot blot assay, we prepared guanine hydrochloride extracted brain homogenates from small cohorts of the following genotypes of 6 month old mice: non-transgenic (non-Tg)/BACE1^−/−^, non-Tg/BACE1^+/+^, 5XFAD/BACE1^−/−^, 5XFAD/BACE1^+/−^, 5XFAD/BACE1^+/+^ (n = 6, except non-Tg/BACE1^−/−^, n = 2). Homogenates were dotted in triplicate onto a nitrocellulose membrane that was incubated with rabbit anti-Aβ42 C-terminal selective antibody followed by goat anti-rabbit-HRP secondary antibody and chemiluminescence detection (Figure 2A). Quantification of dot blot signals revealed that Aβ42 was not detectable in non-Tg/BACE1^−/−^ brain homogenates (Figure 2B), as expected from previous studies [5, 10]. Non-Tg/BACE1^+/+^ homogenates also showed no Aβ42 signal above background, indicating that the dot blot assay is not sensitive enough to detect endogenous mouse Aβ42. 5XFAD/BACE1^−/−^ homogenates had a minor Aβ42 signal that was not significantly different than that for non-Tg/BACE1^+/+^ homogenate. This small 5XFAD/BACE1^−/−^ signal is likely derived from α-secretase cleaved p3 fragment ending in Aβ amino acid 42 (p3(42)) that is overproduced because of transgenic APP overexpression and demonstrates that the anti-Aβ42 C-terminal selective antibody does not recognize full-length APP. 5XFAD/BACE1^+/+^ and 5XFAD/BACE1^+/−^ homogenates both exhibited robust Aβ42 signals with 5XFAD/BACE1^+/−^ having about half the signal of 5XFAD/BACE1^+/+^.

We further assessed the dynamic range of the Aβ42 dot blot technique by comparing 5XFAD/BACE1^+/+^ females at 4, 6 and 9 months (n = 6) to non-Tg/BACE1^+/+^ females at 6 months (n = 6) and to non-Tg/BACE1^−/−^ females (n = 2) (Figure 2C, D). These groups were easily distinguished and signals were detected over at least a ~10-fold dynamic range (between the 4 month and 9 month 5XFAD female mice). Again the non-Tg/BACE1^+/+^ signals were near background, indicating that the dot blot cannot detect endogenous mouse Aβ42.

### Inactivating one BACE1 allele reduces Aβ42 level and amyloidosis in female but not male 5XFAD mice

**Figure 3:**
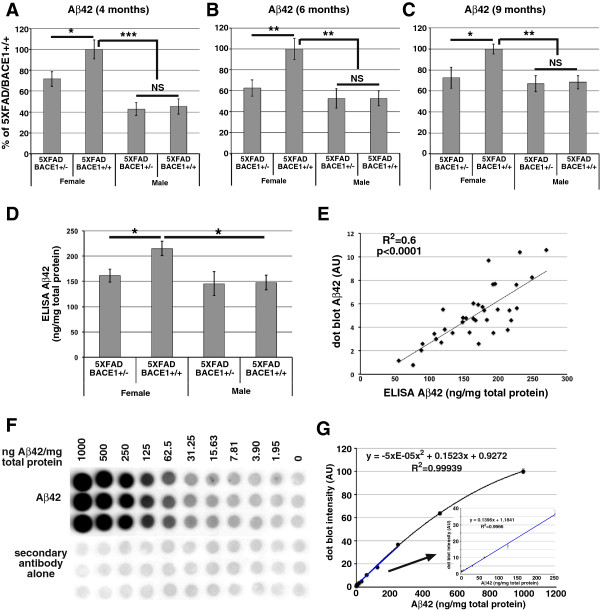
**Aβ42 levels are reduced in female but not male 5XFAD/BACE1** ^**+/−**^ **mice. (A-C)** Aβ42 dot blot assays were performed using GuHCl extracted brain homogenates from 4 **(A)**, 6 **(B)** and 9 **(C)** month-old 5XFAD/BACE1^+/+^ and 5XFAD/BACE1^+/−^ male and female mice (n = 6–17 mice per genotype per sex) and relative quantifications determined and presented as percentages of the mean female 5XFAD/BACE1^+/+^ Aβ42 level. 5XFAD/BACE1^+/−^ females had ~30-40% less Aβ42 than 5XFAD/BACE1^+/+^ females. In contrast, Aβ42 levels of male 5XFAD/BACE1^+/−^ and 5XFAD/BACE1^+/+^ were indistinguishable, and both were significantly less than that of 5XFAD/BACE1^+/+^ females. **(D)** To confirm the relative Aβ42 quantifications generated by the dot blot assay, Aβ42 levels in the brain homogenates from the 6 month-old cohort shown in **(B)** were quantified by commercial Aβ42 ELISA (Invitrogen), yielding very similar results. **(E)** Aβ42 measured by ELISA correlates significantly with Aβ42 measured by dot blot, further validating this method. **(F)** To determine the range and sensitivity of the Aβ42 dot blot assay, known quantities of synthetic Aβ42 were spiked into a transgene-negative BACE1^−/−^ homogenate, which was then GuHCl extracted and analyzed by dot blot (upper blot). Numbers above the blot indicate concentrations of Aβ42 in ng/mg total brain homogenate protein. **(G)** Aβ42 dot blot signals from **(F)** were corrected by subtracting the secondary antibody-alone background, converted to percentage of intensity of 1000 ng Aβ42/mg protein (y-axis), and plotted as a function of known Aβ42 concentration (x-axis). The dot blot is accurate through at least a 500-fold concentration range, with very good linearity from 1.9-250 ng Aβ42/mg total protein, as shown by the blue section of the curve (expanded in the inset). Error bars (SEM) are plotted in both graphs, but are too small to be seen except in the expanded inset.

Next, we used the Aβ42 dot blot assay to assess the effects of BACE1 reduction on cerebral Aβ42 levels in larger cohorts of female and male 5XFAD mice at 4, 6 and 9 months of age (Figure 3A-C; n = 6-17 per sex per genotype per age). We observed a significant ~30-40% reduction in Aβ42 level in 5XFAD/BACE1^+/−^ females compared to 5XFAD/BACE1^+/+^ females at all ages. Surprisingly, male 5XFAD/BACE1^+/−^ mice failed to exhibit any Aβ42 reduction compared to 5XFAD/BACE1^+/+^ males at any age. Notably, female 5XFAD/BACE1^+/+^ mice displayed markedly higher cerebral Aβ42 levels than male 5XFAD mice of either BACE1^+/−^ or BACE1^+/+^ genotypes at all ages, while female 5XFAD/BACE1^+/−^ mice had significantly higher Aβ42 levels than males only at 4 months of age (p = 0.001). These results demonstrate the existence of significant gender differences in the ability of the BACE1^+/−^ genotype to reduce Aβ42 levels in the brains of 5XFAD mice.

We further verified these results by measuring cerebral Aβ42 in the brains of the 6-month old mice by commercial Aβ42 sandwich ELISA (Figure 3D). As before, we noted a significant ~30% decrease in Aβ42 in 5XFAD/BACE1^+/−^ compared to 5XFAD/BACE1^+/+^ females, but no difference between 5XFAD/BACE1^+/−^ and 5XFAD/BACE1^+/+^ males, corroborating our Aβ42 dot blot results. Aβ42 levels measured by dot blot correlated well with Aβ42 measured by ELISA (Figure 3E), further validating the dot blot assay as a simple, inexpensive, and accurate technique for relative quantifications of cerebral Aβ42.

To determine the sensitivity and dynamic range of the dot blot method, synthetic human Aβ42 was resuspended in DMSO, diluted in 4 mM HEPES, and spiked into a brain homogenate from a transgene-negative BACE1^−/−^ mouse (which lacks endogenous Aβ) at 2-fold concentration steps, always maintaining total protein concentration at 10 mg/ml. These preparations were then extracted in GuHCl and subjected to Aβ42 dot blot analysis as for the 5XFAD brain homogenates (Figure 3F). We observed that the dot blot assay remains accurate and nearly linear over at least a 500-fold range from 1.9 to 1000 ng of synthetic Aβ42/mg total protein (Figure 3G). When intensity is plotted as a function of Aβ42 concentration from 1.9 to 1000 ng Aβ42, the curve is best fit by a second order polynomial function. We have used the same dot blot method to measure Aβ42 ranging from 1.5-30 ng/mg total protein in brain homogenates of cognitively normal, aged humans [29], and ranging from 50–275 ng/mg total protein in 5XFAD brain homogenates (Figure 3E). In these ranges, the assay shows a very linear (R^2^ = 0.9966) relationship between Aβ42 concentration and dot blot signal intensity (Figure 3G, inset). Thus, the dot blot assay is sensitive and linear in the range needed to measure Aβ42 in the brains of 5XFAD mice, other mouse models of AD, and aged humans.

**Figure 4:**
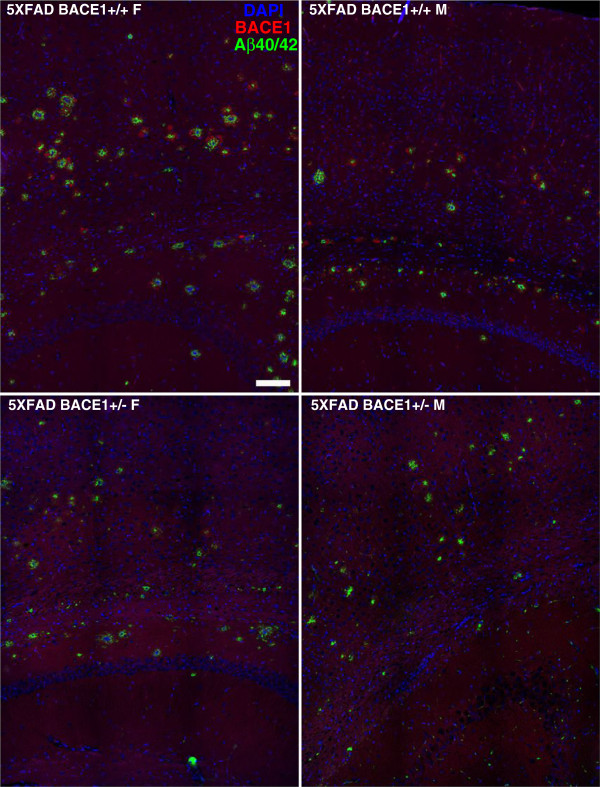
**Amyloid plaques are reduced in female but not male 5XFAD/BACE1** ^**+/−**^ **mice.** Free-floating 30 μm brain sections from 6 month-old male and female 5XFAD/BACE1^+/+^ and 5XFAD/BACE1^+/−^ mice were co-stained with clone 3D6, an antibody to the N-terminus of Aβ (green), anti-BACE1 antibody (red), and DAPI (blue) and imaged by fluorescence confocal microscopy. Three mice of each sex and genotype combination were analyzed for a total of twelve mice, and all images were captured using identical confocal camera and laser configurations. The representative images of mid-dorsal cortex and hippocampus shown here are from individuals that had Aβ42 dot blot values near the mean of each group. Amyloid plaque densities in Layer 5 of the cortex correlate well with Aβ42 levels determined by dot blot. Female 5XFAD/BACE1^+/+^ mice have the highest plaque density, with 5XFAD/BACE1^+/−^ females showing a marked reduction in amyloid deposits by comparison. In contrast, male 5XFAD/BACE1^+/+^ and 5XFAD/BACE1^+/−^ mice had similar plaque densities, and both had fewer plaques than 5XFAD/BACE1^+/+^ female mice. These histological results support our biochemical data showing that Aβ42 levels are reduced in 5XFAD/BACE1^+/−^ female, but not male, mice (Figure 3). Size bar represents 100 μm.

Based on the biochemical findings described above, we anticipated a decrease in the Aβ42 plaque load in 5XFAD/BACE1^+/−^ females compared to 5XFAD/BACE1^+/+^ females, and in both the 5XFAD/BACE1^+/+^ and 5XFAD/BACE1^+/−^ males compared to 5XFAD/BACE1^+/+^ females. To investigate this, we co-immunostained brain sections from 6-month old male and female mice of the above genotypes with antibodies against Aβ (clone 3D6) and BACE1, then imaged the sections by immunofluorescence confocal microscopy (Figure 4). As expected, qualitative inspection of representative images indicated that female 5XFAD/BACE1^+/−^ mice have a reduction in size and number of amyloid plaques throughout Layer 5 cortex and hippocampus compared to female 5XFAD/BACE1^+/+^ mice. As suggested by Aβ42 dot blot and ELISA measurements, 5XFAD/BACE1^+/+^ and 5XFAD/BACE1^+/−^ male mice appeared to have similar sizes and numbers of plaques and both have many fewer plaques than 5XFAD/BACE1^+/+^ females. BACE1 immunostaining was visible in a halo of dystrophic neurites around plaques in all 5XFAD mice [28, 30], although peri-plaque BACE1 fluorescence intensity was noticeably reduced in 5XFAD/BACE1^+/−^ mice of both genders. These data suggest that 50% reduction in BACE1 level results in less BACE1 accumulation in dystrophic neurites around plaques, and is associated with reduced amyloid deposition, at least in female 5XFAD/BACE1^+/−^ mice.

Taken together, our biochemical and immunofluorescence microscopy results indicate that 50% BACE1 reduction in 5XFAD mice decreases cerebral Aβ42 level and amyloid plaque load in female, but not male, mice. Additionally, female 5XFAD mice exhibit higher levels of Aβ42 and plaques compared to males, at least for the 5XFAD/BACE1^+/+^ genotype.

### Relative levels of BACE1-cleaved APP fragments sAPPβ and C99 frequently parallel levels of Aβ42 and amyloid deposition in 5XFAD/BACE1^+/+^ and 5XFAD/BACE1^+/−^ mice

**Figure 5:**
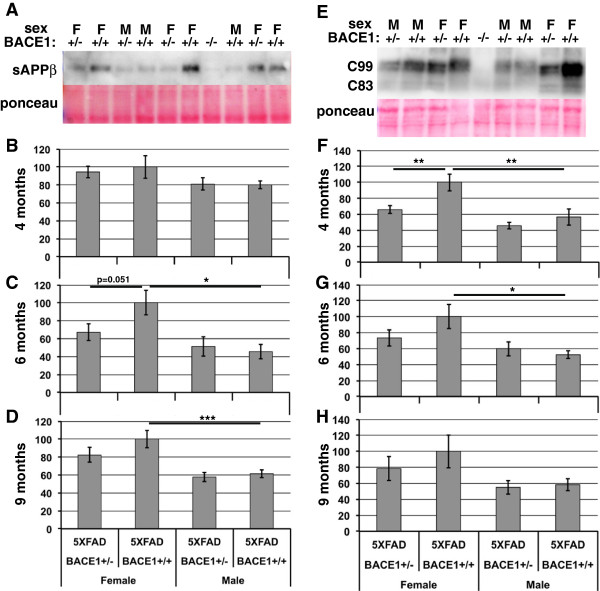
**BACE1-cleaved APP fragments sAPPβ and C99 are reduced in female but not male 5XFAD/BACE1** ^**+/−**^ **mice. (A)** Representative sAPPβ immunoblot and Ponceau S staining of brain homogenates from 4 month-old male (M) and female (F) mice. Note the minimal sAPPβ signal in the 5XFAD/BACE1^−/−^ lane; residual band likely results from minor cross-reactivity to sAPPα. **(B-D)** Relative quantifications of sAPPβ immunoblot signals from 5XFAD/BACE1^+/+^ and 5XFAD/BACE1^+/−^ mice. At 4 months of age there is no statistical difference in sAPPβ levels between the sexes or genotypes. However, at 6 and 9 months of age 5XFAD/BACE1^+/−^ females show trends toward lower sAPPβ levels than 5XFAD/BACE1^+/+^ females, mirroring the significant reduction in Aβ42 levels. sAPPβ levels in 6 and 9 month-old 5XFAD/BACE1^+/−^ and 5XFAD/BACE1^+/+^ male are indistinguishable, and significantly less than in 5XFAD/BACE1^+/+^ females. **(E)** Representative of C99 immunoblot and Ponceau S staining of brain homogenates from 9 month-old male (M) and female (F) mice. The doublet bands reflect the phosphorylation state of the APP C-terminal fragments. The lower doublet represents the α-secretase-cleaved APP C-terminal fragment C83. Note the absence of the C99 signal in the 5XFAD/BACE1^−/−^ lane. **(F-H)** Relative quantification of C99 immunoblot signals from male and female 5XFAD/BACE1^+/+^ and 5XFAD/BACE1^+/−^ mice. At all ages, 5XFAD/BACE1^+/−^ females have lower levels of C99 than 5XFAD/BACE1^+/+^ females, but this difference reaches statistical significance only at 4 months. In contrast, C99 levels are not significantly different between 5XFAD/BACE1^+/+^ and 5XFAD/BACE1^+/−^ males at any age. At 4 and 6 months of age, 5XFAD/BACE1^+/+^ males have significantly less C99 than 5XFAD/BACE1^+/+^ females, but at 9 months the decrease does not reach statistical significance due to large variation in the female cohort. Overall, the patterns of sAPPβ and C99 levels correlate with those of Aβ42 levels for the different genotypes and genders.

We wished to gain further insight into the basis of the difference in Aβ42 levels between 5XFAD/BACE1^+/+^ males and females, and the lack of effect of BACE1 heterozygosity on Aβ42 in males, by measuring the direct products of BACE1 cleavage of APP: the ~100 kDa secreted N-terminal fragment, sAPPβ, and ~11 kDa membrane-bound C-terminal fragment C99. Differences in levels of cerebral Aβ42 and amyloid plaques could arise from variations in Aβ42 production, clearance, or both. Parallel patterns of relative levels of Aβ42, sAPPβ, and C99 in 5XFAD brains would support the conclusion that differences in Aβ42 levels are the result of variations in BACE1 cleavage of APP, not Aβ42 clearance. In contrast, divergent patterns of Aβ42, sAPPβ, and C99 levels could indicate different degrees of Aβ42 clearance without changes in Aβ42 production rates. To investigate these alternative scenarios, we quantified levels of sAPPβ and C99 via immunoblot analysis (Figure 5, n = 6–17 per sex per genotype per age). In general, the patterns of levels for both sAPPβ and C99 were similar to those seen for Aβ42 for the different genotypes, genders, and ages of 5XFAD mice. A trend toward reduced levels of sAPPβ were observed at 6 and 9 months of age in female 5XFAD/BACE1^+/−^ compared to female 5XFAD/BACE1^+/+^, but these differences failed to reach statistical significance (Figure 5C and D). As with Aβ42, no difference in sAPPβ level was seen between males of either genotype. Again like Aβ42, 5XFAD/BACE1^+/+^ and 5XFAD/BACE1^+/−^ males exhibited a significant ~40-50% reduction in sAPPβ compared to 5XFAD/BACE1^+/+^ females at 6 and 9 months of age. Unlike Aβ42, no significant differences in sAPPβ levels were observed between the different genotypes or genders of 5XFAD mice at 4 months of age. Results similar to those of sAPPβ and Aβ42 were obtained for levels of C99 in 5XFAD brains (Figure 5F-H). We observed that female 5XFAD/BACE1^+/−^ have lower C99 levels than female 5XFAD/BACE1^+/+^ mice. Athough non-significant trends were seen at 6 and 9 months, the C99 decrease was statistically significant at 4 months (Figure 5E-H). Also recapitulating the pattern of sAPPβ and Aβ42, there was no difference between C99 levels in 5XFAD/BACE1^+/−^ and 5XFAD/BACE1^+/+^ males, but both have on average only ~50% of the C99 level in 5XFAD/BACE1^+/+^ females.

Although some of the differences in sAPPβ and C99 between 5XFAD/BACE1^+/+^ and 5XFAD/BACE1^+/−^ females failed to reach statistical significance, there were decreases in both cleavage products at all time-points in the female 5XFAD/BACE1^+/−^, except for 4 month sAPPβ levels. In contrast, male 5XFAD/BACE1^+/−^ mice never showed reductions in sAPPβ and C99 compared to male 5XFAD/BACE1^+/+^ mice. Because sAPPβ and C99 immunoblots were only semi-quantitative and had notable background leading to high variability, statistical significance was not achieved in some cases. In contrast, Aβ42 dot blots had much lower background and each sample was measured in triplicate, greatly reducing variability and improving the ability to achieve statistically significant results. When both significant differences and non-significant trends were considered, the general patterns of relative quantifications of Aβ42, sAPPβ, and C99 paralleled one another quite well. While it remains possible that differences in Aβ42 levels in our 5XFAD mice result from altered clearance rates or other mechanisms, we interpret these data to suggest that the reduced Aβ42 level of 5XFAD/BACE1^+/−^ females compared to 5XFAD/BACE1^+/+^ females is likely due to decreased BACE1 cleavage of APP. Similarly, the reduced C99 and sAPPβ in 5XFAD/BACE1^+/+^ males compared to 5XFAD/BACE1^+/+^ females suggest that the lower levels of Aβ42 and amyloid deposition in 5XFAD/BACE1^+/+^ males is also due to decreased BACE1 cleavage of APP. Since BACE1 levels were similar in males and females of the same genotype at 4, 6 and 9 months (Figure 1), we hypothesized that APP transgene expression may vary between male and female 5XFAD mice.

### Transgenic APP level is increased and correlates with elevated Aβ42 in 5XFAD female mice

**Figure 6:**
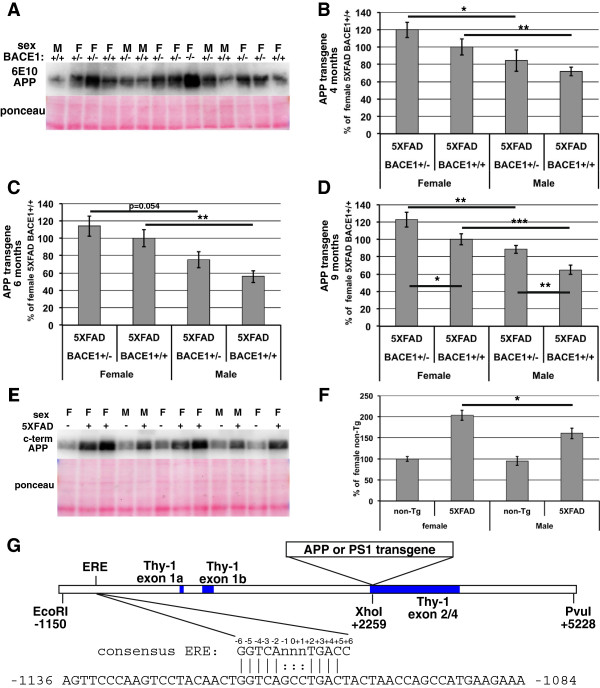
**Female 5XFAD mice of either BACE1** ^**+/+**^ **or BACE1** ^**+/−**^ **genotypes have higher steady-state transgenic APP levels than males. (A)** Representative transgenic APP immunoblot (6E10) and Ponceau S staining of brain homogenates from 4 month old male (M) and female (F) mice. Note the high level of APP in the 5XFAD/BACE1^−/−^ lane. **(B-D)** Relative quantifications of transgenic APP immunoblot signals. At all ages, steady-state transgenic APP levels are higher in 5XFAD/BACE1^+/−^ and 5XFAD/BACE1^+/+^ females than in corresponding males. Additionally, there is a trend toward higher transgenic APP levels in 5XFAD/BACE1^+/−^ compared to 5XFAD/BACE1^+/+^ mice that becomes significant at 9 months, which could result from less BACE1 cleavage in the BACE1^+/−^ genotype. **(E)** Representative APP immunoblot (C-terminal APP antibody) and Ponceau S staining of brain homogenates from 6 month old 5XFAD/BACE1^+/+^ (+) and non-Tg/BACE1^+/+^ (−) mice to measure both human and mouse APP. **(F)** Relative quantifications of APP immunoblot signals in **(E)** demonstrates that while non-Tg males and females have equal levels of endogenous APP, 5XFAD females have significantly higher APP levels than 5XFAD males, a result that is consistent with the 6E10 immunoblot data showing elevated transgenic APP levels in 5XFAD females. n = 9–14 mice per sex per genotype. **(G)** Schematic diagram of murine Thy-1 promoter transgene cassette with 5’ upstream sequence showing estrogen resonse element (ERE). The APP or PS1 coding regions are inserted into the XhoI site. Thy-1 gene exons are shaded blue. Base-pair (bp) numbering is relative to the transcriptional start site for Thy-1 exon 1a [32]. The ERE is located from −1116 bp to −1104 bp in the 5’ upstream regulatory region of the Thy-1 promoter. The one bp deviation of the Thy-1 ERE at position +6 could be overcome by the flanking A-T rich sequence, which enhances ERE transcriptional potency *in vivo*[33].

One potential reason for increased cerebral Aβ42 accumulation, and increased levels of APP cleavage products C99 and sAPPβ in female 5XFAD mice could involve higher female expression of the APP transgene. To investigate this possibility, we performed immunoblot analysis with an anti-APP antibody specific to human APP (clone 6E10) to measure the steady-state levels of transgenic APP in brain homogenates from the same 5XFAD mice analyzed for Aβ42 by dot blot assay (Figure 6A-D, n = 6–17 per sex per genotype per age). We observed that at all ages 1) female 5XFAD/BACE1^+/+^ mice have ~30-40% higher levels of APP than male 5XFAD/BACE1^+/+^, 2) female 5XFAD/BACE1^+/−^ mice exhibit ~30-40% higher levels of APP than male 5XFAD/BACE1^+/−^, and 3) APP levels in 5XFAD/BACE1^+/−^ mice are significantly higher than those in 5XFAD/BACE1^+/+^ mice for both genders. The first two findings demonstrate that transgenic APP levels are markedly elevated in female compared to male 5XFAD mice when we control for BACE1 genotype. The third finding is consistent with the hypothesis that 50% BACE1 level in 5XFAD/BACE1^+/−^ mice results in reduced BACE1 cleavage of APP that causes elevated steady-state APP levels, and that α-secretase processing of APP cannot fully compensate for partial BACE1 reduction. This principle is demonstrated most dramatically in 5XFAD/BACE1^−/−^ mice in which there is no BACE1 cleavage of APP and steady-state APP levels are at their highest [6] (Figure 6A).To verify our APP immunoblot results obtained with the 6E10 antibody, we estimated the levels of transgenic APP compared to endogenous APP using an antibody that recognizes the C-terminus of both mouse and human APP (Figure 6E). When brain homogenates from 6-month old non-Tg and 5XFAD mice (n = 9–14 per sex per genotype) were subjected to immunoblot analysis with the APP C-terminal antibody, we found that female 5XFAD mice have ~200% of the APP level found in non-Tg females, while male 5XFAD mice have ~160% of the female non-Tg APP level (Figure 6F). In contrast, there was no significant difference in levels of endogenous APP between non-Tg males and females. When the immunoblot signal representing endogenous APP is subtracted, female 5XFAD mice have ~40% higher steady-state transgenic APP levels than 5XFAD males, in good agreement with the female ~30-40% elevations observed with the human APP-specific antibody 6E10. We conclude that the increased steady-state level of transgenic APP in female 5XFAD mice likely has a role in elevated cerebral Aβ42 accumulation in 5XFAD females compared to males.

The difference in transgenic APP level observed between male and female 5XFAD mice could be due to gender differences in APP transgene transcription or translation, mRNA or protein stability, or some post-translational modification or other process. Since steady-state levels of endogenous APP do not appear to differ between male and female non-Tg mice, it appeared unlikely that gender-specific alterations in expression or stability exist, at least for endogenous APP. Therefore, we reasoned that the higher 5XFAD female transgenic APP level is more likely the result of a gender difference in transgene expression due to an effect of the murine Thy-1 promoter cassette [31] that drives APP transgene transcription, or from the chromosomal locus into which the transgene has integrated. If the transgene Thy-1 promoter is responsible, then one might predict the presence of an estrogen responsive element (ERE) in the 5′ upstream regulatory region of the 5XFAD transgene. Indeed, inspection of the Thy-1 regulatory sequence found in the Thy-1 transgene cassette (GenBank accession AC126459.3, nucleotides 7732–11902, reverse complement) revealed an ERE at base pairs −1116 to −1104 relative to the transcriptional start site of Thy-1 exon 1a [32], however this ERE exhibited a single nucleotide change from the consensus ERE at position +6 (Figure 6G). Although the Thy-1 promoter ERE is imperfect, it is surrounded by A-T rich sequence, which is predicted to increase ERE transcriptional activity [33]. Thus, although we cannot presently exclude other potential mechanisms, the presence of the ERE in the APP transgene promoter is likely to explain elevated transgenic APP levels in female compared to male 5XFAD mice.

## Discussion

Here, we report that 50% BACE1 reduction lowers cerebral Aβ42 levels by ~30-40% in female, but not male, 5XFAD/BACE1^+/−^ mice. Similarly, 50% BACE1 reduction lowers sAPPβ and C99 levels in female 5XFAD/BACE1^+/−^ mice, paralleling the Aβ42 decrease and suggesting reduced BACE1 cleavage of APP, although other mechanisms such as increased Aβ42 clearance remain formally possible. We also show that female 5XFAD mice have higher cerebral Aβ42 levels than males, which is most pronounced in the BACE1^+/+^ genetic background and correlates with higher steady state transgenic APP levels in female compared to male 5XFAD mice. Importantly, an ERE exists in the neuron-specific 5XFAD transgene murine Thy-1 promoter sequence, which potentially could explain the increased transgenic APP level and, in turn, the higher cerebral Aβ42 accumulation in female 5XFAD mice [27]. Finally, we report a robust and accurate Aβ42 dot blot assay for measuring relative Aβ42 levels in the brain as a cost-effective alternative to Aβ42 ELISA. Together, these results reveal that the effectiveness of 50% BACE1 inhibition in reducing cerebral Aβ42 is affected by relative levels of BACE1 and APP, which has important implications for use of BACE1 inhibition in preventing or treating AD.

We have previously reported that our dot blot assay can measure Aβ42 in the brains of cognitively normal, aged humans, with results comparable to those generated by ELISA [29]. By ELISA, Aβ42 varied from 1.5-30 ng/mg total protein in homogenates of normal human brains, which is in the linear range of our dot blot method. Here we demonstrate that the dynamic range of the Aβ42 dot blot assay is at least 500 fold, and it is sensitive and linear in the range required for measuring Aβ42 in the 5XFAD mouse model as well (Figure 3G). This dot blot assay can be easily adapted to measure absolute Aβ42 levels by including known quantities of synthetic Aβ42 on the dot blot for generating a standard curve. The range of the standards for a commonly used commercial Aβ42 sandwich ELISA (e.g., Wako, Inc.) is 4-400 pg/ml, while the standards we used for the dot blot assay range from 7.7-3900 ng/ml. While the ELISA has the ability to detect very low concentrations of Aβ42, as with plasma or cerebral spinal fluid samples, this is not necessarily advantageous for samples with high Aβ42 concentrations, such as brain homogenates of APP transgenic mice or aged humans, because they have to be diluted up to 5000-fold, leaving room for pipetting error, while the dot blot assay requires little if any dilution, and is thus potentially less error-prone. Additionally, the linear range of the ELISA readout is only around 100-fold, while our dot blot is 500 fold, allowing the comparison of diverse samples. In short, we suggest the dot blot technique for measuring Aβ42 levels in brain homogenates of mouse models of AD and in human subjects, though it is likely to be less sensitive for measuring low concentrations of Aβ42 in samples such as cerebral spinal fluid or plasma.

The findings reported here are congruous with those of other studies showing reduced Aβ and amyloid pathology in APP transgenic mice with one inactive allele of BACE1 [5, 14, 21–26]. The degree of Aβ lowering with 50% BACE1 reduction varies depending on the study, which might be related to differences in the APP transgenic mouse models employed, such as APP mutation, overexpression level, and strain background, among others. The level of Aβ lowering in female 5XFAD/BACE1^+/−^ mice in our study was most similar to that observed in APPSwe/PS1E9/BACE1^+/−^ mice, in which Aβ42 levels are decreased by 40% and 27% at 3 and 12 months, respectively [14].

Our observation that 50% BACE1 reduction in the 5XFAD transgenic line decreases Aβ42 levels in females only was unexpected. Previous studies of BACE1^+/−^ mice on various APP transgenic backgrounds reported no differences in Aβ levels between males and females [14, 26] or did not analyze the genders separately [21–24]. The reason for the lack of Aβ42 lowering in male 5XFAD/BACE1^+/−^ mice remains enigmatic, although it might involve the lower level of expression of the human APP transgene in male compared to female 5XFAD mice. We hypothesize that even with 50% BACE1 reduction in male 5XFAD/BACE1^+/−^ mice, BACE1 levels are still sufficiently in excess of those of APP in the intracellular compartments where β-secretase processing occurs such that the same amount of APP can be cleaved as in male 5XFAD/BACE1^+/+^ mice. In contrast, in female 5XFAD/BACE1^+/−^ mice, BACE1 levels might not be in excess of those of APP because of the higher female expression of the APP transgene. This could render BACE1 rate-limiting, so that at the 50% level BACE1 cleaves less APP within β-secretase intracellular compartments, leading to a reduction in Aβ42, C99, and sAPPβ in 5XFAD/BACE1^+/−^ compared to 5XFAD/BACE1^+/+^ females. This notion is supported by studies showing that PDAPP/BACE1^+/−^ mice with very high (~10 fold) APP transgene expression [34] have 90% reduction in Aβ at 13 months and 50% reduction at 18 months [24], while decreases in endogenous Aβ are small in non-Tg BACE1^+/−^ mice lacking an overexpressed APP transgene [25, 26]. However, contrary to the above argument, we also observed that in both males and females, full-length transgenic APP levels are significantly increased in 5XFAD/BACE1^+/−^ compared to 5XFAD/BACE1^+/+^ mice at 9 months of age, and trend higher at the younger ages, regardless of gender, indicating reduced β-secretase cleavage of APP in 5XFAD/BACE1^+/−^ of both sexes. Thus, there may be additional factors other than transgenic APP level causing the difference in Aβ lowering between male and female 5XFAD/BACE1^+/−^ mice.

These data, along with our current findings suggest that the degree to which BACE1 activity must be inhibited to significantly reduce cerebral Aβ42 levels will depend on whether BACE1 is in excess of APP and by how much. Depending of the study, in wild type mice, where APP is not overexpressed, BACE1 heterozygosity has either no effect on Aβ40 or Aβ42 [25] or reduces Aβ40 by ~10% [26]. In AD patients, BACE1 is elevated in dystrophic neurites surrounding plaques, as is APP [28, 30], so it difficult to determine whether BACE1 is in excess of APP or not. BACE1 elevation in AD brains is detectable by immunoblot and by enzymatic activities [28, 30, 35, 36] but APP elevation by immunoblot has not been reported. The elevation of BACE1 protein levels and enzymatic activity in AD brains suggests that BACE1 could be in excess of APP, so we predict that BACE1 inhibition greater than 50% will be required to significantly reduce cerebral Aβ42 levels in AD. While BACE1 inhibitors in clinical trials are able to lower cerebral spinal fluid Aβ by ~50-90 % indicating highly effective BACE1 inhibition (reviewed in [9]), concerns about mechanism-based side effects related to BACE1 over-inhibition exist [8]. BACE1^−/−^ mice have multiple complex but subtle neurological abnormalities, though it is not yet clear whether these stem from the absence of BACE1 during crucial developmental periods, or during adult life.

It should be noted that the presence of the APP Swedish mutation in the 5XFAD mouse line could also affect the degree to which 50% BACE1 reduction lowers Aβ42 generation, although probably not dramatically. This mutation makes APP a better substrate for BACE1 [37] so a larger proportion of APP molecules become cleaved to produce Aβ. Rabe et al. [26] showed that heterozygous BACE1 gene deletion reduces Aβ levels by 16% in mice expressing a wild-type APP transgene, which more likely reflects the situation in sporadic AD, and by 20% in mice expressing an equivalent level of transgenic APP with the Swedish mutation. Although 50% BACE1 reduction lowered Aβ levels in Swedish mutation APP mice greater than in wild-type APP mice by a modest amount, these results suggest the Swedish mutation does not dramatically alter the effect of partial BACE1 reduction on Aβ generation, at least in the mouse strains studied [26].

Our study furthers the characterization of the 5XFAD mouse model, which is now widely used by the AD research community, and highlights the variation between males and females in this line. Differences between males and females in Aβ levels and plaque deposition have been reported in other mouse models of AD such as the Tg2576, 3xTg-AD, and APPswe/PS1de9 lines [38–40], and may be related to estrogen levels [41]. These results mirror observations in the human population that AD risk is higher for females than males, even after correcting for increased longevity [42], but it is still not clear why women are more susceptible. Our observation that female 5XFAD mice have higher steady-state levels of transgenic APP compared to males is likely responsible for increased female 5XFAD Aβ42 level and amyloid deposition and appears to be related to the presence of an ERE in the 5′ upstream regulatory region of the murine Thy-1 transgene promoter. Levels of endogenous APP did not differ between male and female non-Tg mice, suggesting that female humans do not have higher cerebral APP expression. Our results caution that the 5XFAD mouse and other AD transgenic models that employ the Thy-1 promoter are not appropriate models for the gender disparity observed in AD. Thus, a mechanism other than increased steady-state APP level is likely responsible for the higher incidence of AD in women. Additionally, these results illustrate the critical importance of designing studies in which the control and experimental groups contain equal numbers of males and females, and that data from the two genders should be analyzed separately to determine differential effects of the experimental condition, and in order to avoid false gender-specific differences. If gender effects are observed in this model, or others using the Thy-1 promoter, it is important to determine whether the cause may be related to different levels of transgene expression.

## Conclusions

In summary, 50% BACE1 reduction in 5XFAD mice decreases cerebral Aβ42, plaque load, and BACE1-cleaved fragments of APP in females but not males. Female 5XFAD mice express higher levels of transgenic APP, probably because of an ERE in the Thy-1 transgene promoter, which in turn likely explains elevated female Aβ42. As a consequence of higher transgenic APP level, we hypothesize that BACE1 level is not in excess of that of APP in female 5XFAD mice, so that 50% BACE1 reduction significantly decreases Aβ42. In contrast, BACE1 could be in excess of APP in male 5XFAD mice, and as a result 50% BACE1 reduction has little effect on lowering Aβ42 levels. Since BACE1 is likely to be in excess of APP in the human brain, we suggest that partial BACE1 inhibition should be greater than 50% to have therapeutic benefit for AD. Finally, our Aβ42 dot blot assay offers an accurate, simple, and cost-effective alternative to expensive commercial Aβ42 ELISAs.

## Methods

### Mice

5XFAD mice overexpress the K670N/M671L (Swedish), I716V (Florida), and V717I (London) mutations in human APP(695), as well as M146L and L286V mutations in human PS1. The generation of the 5XFAD mice has been described previously [27]. These mice were crossed to a BACE1^−/−^ line generated as described [4, 5]. All mice were genotyped by PCR amplification of tail clip DNA. Mice were euthanized by carbon dioxide inhalation. One hemibrain was snap-frozen in liquid nitrogen. The other hemibrain was drop fixed in 4% paraformaldehyde in PBS for 16–20 hrs, then transferred to 30% w/v sucrose in 1×PBS with azide for storage. All animal work was done in accordance with Northwestern University IACUC approval. The numbers of mice per age per sex per genotype are as follows: 4 month female 5XFAD/BACE1^+/−^: 17; 4 month female 5XFAD/BACE1^+/+^: 7; 4 month male 5XFAD/BACE1^+/−^: 10; 4 month male 5XFAD/BACE1^+/+^: 12; 6 month female 5XFAD/BACE1^+/−^: 14; 6 month female 5XFAD/BACE1^+/+^: 9; 6 month male 5XFAD/BACE1^+/−^: 6; 6 month male 5XFAD/BACE1^+/+^: 12; 9 month female 5XFAD/BACE1^+/−^: 11; 9 month female 5XFAD/BACE1^+/+^: 13; 9 month male 5XFAD/BACE1^+/−^: 10; 9 month male 5XFAD/BACE1^+/+^: 16.

### Immunoblots and ELISA

Snap frozen hemi-brains were homogenized in 1 ml 1×PBS with 1% Triton X-100 supplemented with protease inhibitors (Calbiochem) and Halt Phosphatase Inhibitor Cocktail (Thermo Scientific). Protein concentration was quantified using BCA Assay (Pierce). Twenty micrograms of brain homogenate were separated on 12% Tris-glycine gels and protein was transferred to 0.45 μm PVDF membrane. For C99, proteins were separated on 16% Tris-Tricine gels using cathode buffer containing 200 mM Tris, 200 mM Tricine, 0.2% SDS, pH 8.3 and anode buffer containing 100 mM Tris HCl, pH 8.9. Membranes were Ponceau S-stained immediately after transfer, then probed overnight with anti-BACE1 antibody (BACE-Cat1, Vassar lab, 1:1000) [28], anti-APP (6E10, Covance #SIG-39320-500 1:2000) anti-APP (C-terminal rabbit monoclonal, Epitomics #1565-1, 1:5000) anti-sAPPβ (Beta-APPsw 1:1000, Vassar et al., 1999) in 5% milk, followed by washing and a 1 hour incubation with secondary HRP-conjugated anti-mouse or anti-rabbit secondary antibody (Vector Labs, PI-2000 or PI-1000, 1:10,000). Blots were visualized using Luminata Crescendo (Millipore), and signals were quantified using a Kodak Image Station 4000R. All signals were normalized to Ponceau S staining. For theses analyses, multiple gels were cut into horizontal strips and stacked so all samples for a given protein were transferred to a single piece of PVDF membrane. Putting all samples (up to 50) on one membrane eliminated the need to account for variation in transfer, antibody incubation and ECL application that can occur between blots. Student’s two-tailed t-test were done using InStat software (GraphPad Software, Inc., San Diego, CA) to compare the following pairs: 5XFAD/BACE1^+/+^ females to 5XFAD/BACE1^+/−^ females, 5XFAD/BACE1^+/+^ females to 5XFAD/BACE1^+/+^ males, and 5XFAD/BACE1^+/+^ males to 5XFAD/BACE1^+/−^ males. For the bar graphs, lines with stars indicate groups statistically compared by Student’s t-test; * 0.05 > p > 0.01 ** 0.01 > p > 0.001 *** 0.001 > p > 0.0001; Error bars = S.E.M.

For Aβ42 dot blots, 10 mg/ml brain homogenates were extracted in 1.56 volumes of 8.2 M guanidine hydrochloride (GuHCl); 82 mM Tris HCl (pH 8.0) (5 M GuHCl final) over one to three nights on a nutator. To create the Aβ42 standard curve, HFIP lyophilized Aβ42 (rPeptide) was resuspended at 5 mM in DMSO, then diluted to 100 μM in 4 mM HEPES, and spiked into 10 mg/ml transgene negative BACE1^−/−^ brain homogenates at 2-fold concentration steps ranging from 1.95 to 100 ng Aβ42/mg total protein. Standard curve samples were then GuHCl extracted as described. For dot blots, 1 μl of GuHCl extracted sample (3.9 μg total protein) was spotted in triplicate on gridded nitrocellulose membrane, dried one hour at 37°C, then stained with Ponceau S. Two identical blots were made and incubated in either 1:2500 anti-Aβ42 rabbit monoclonal antibody (clone H31L21, Invitrogen, #700254) or 5% milk only (primary delete), followed by HRP-conjugated secondary antibody (Vector Labs, PI-1000). The duplicate blots incubated with anti-Aβ42 antibody or primary delete were developed together with Luminata Crescendo (Millipore) or West Femto (Pierce) for standard curve, and imaged simultaneously using the Kodak Image Station 4000R or MyECL Imager (Thermo Scientific). No images with saturated pixels were used for quantification. Aβ42 signals were normalized to Ponceau S staining, and the triplicates averaged, and statistics were performed as described above. For ELISA, GuHCl extracted samples were diluted 1:1000, and Aβ42 ELISA performed according to manufacturer’s instructions (Invitrogen). Standard curve equations were generated in Excel, using intensities from images with no saturated pixels. For the bar graphs, lines with stars indicate groups statistically compared by Student’s t-test; * 0.05 > p > 0.01 ** 0.01 > p > 0.001 *** 0.001 > p > 0.0001; Error bars = S.E.M.

### Immunofluorescence confocal microscopy

Paraformaldehyde-fixed brains were sectioned at 30 μm. Free-floating sagittal (females) or coronal (males) sections were treated with TBS-0.25% Triton X-100 with 16 mM glycine for 60 minutes on a shaker. Sections were washed in TBS, blocked in 5% donkey serum in TBS-0.25% Triton X-100 for one hour, then washed 2x10 minutes in 1% BSA in TBS-0.25% Triton X-100. Sections were incubated at 4°C on a shaker overnight with anti-Aβ 3D6 (mouse monoclonal, 1:250, gift from Lisa McConlogue, Elan). The following morning, anti-BACE1 (1:250, rabbit monoclonal, Abcam ab108394) was added and the sections were incubated an additional 2 hours at 37°C with shaking. The sections were washed 3 times with TBS then incubated for 1.5 hours at room temperature on a shaker with Invitrogen Alexa Fluor secondary antibodies at 1:250 (Donkey anti-Rabbit 594 and donkey anti-mouse 488) and DAPI at 300 nM. The sections were then mounted on slides and immediately cover-slipped using ProLong Gold (Invitrogen). Confocal images were captured on a Nikon (Tokyo, Japan) A1R confocal microscope with a 40x objective, and 12 images were stitched together using NIS Elements software to generate a larger field image. All images were captured at same laser and software configurations.

## Abbreviations

BACE1: β-site APP cleaving enzyme 1
APP: Amyloid precursor protein
Aβ42: β-amyloid 42 peptide
AD: Alzheimer’s disease.
